# Parallel Thalamic Pathways for Whisking and Touch Signals in the Rat

**DOI:** 10.1371/journal.pbio.0040124

**Published:** 2006-04-18

**Authors:** Chunxiu Yu, Dori Derdikman, Sebastian Haidarliu, Ehud Ahissar

**Affiliations:** **1**Department of Neurobiology, The Weizmann Institute of Science, Rehovot, Israel; Vanderbilt UniversityUnited States of America

## Abstract

In active sensation, sensory information is acquired via movements of sensory organs; rats move their whiskers repetitively to scan the environment, thus detecting, localizing, and identifying objects. Sensory information, in turn, affects future motor movements. How this motor-sensory-motor functional loop is implemented across anatomical loops of the whisker system is not yet known. While inducing artificial whisking in anesthetized rats, we recorded the activity of individual neurons from three thalamic nuclei of the whisker system, each belonging to a different major afferent pathway: paralemniscal, extralemniscal (a recently discovered pathway), or lemniscal. We found that different sensory signals related to active touch are conveyed separately via the thalamus by these three parallel afferent pathways. The paralemniscal pathway conveys sensor motion (whisking) signals, the extralemniscal conveys contact (touch) signals, and the lemniscal pathway conveys combined whisking–touch signals. This functional segregation of anatomical pathways raises the possibility that different sensory-motor processes, such as those related to motion control, object localization, and object identification, are implemented along different motor-sensory-motor loops.

## Abstract 2

Ahissar and colleagues provide novel evidence that whisking and touch signals related to whisker stimulation in rats are conveyed by three parallel pathways in the thalamus.

## Introduction

Active touch is a closed-loop process in which sensor motion determines the sensory input and the sensory input determines future sensor motion [ 1, 2]. Both sensor motion and touch signals are reported to the brain by peripheral neurons. Limb movements are reported via proprioceptive mechanoreceptors located in joints, tendons, and muscle spindles. Whisker movements are reported to the brain via mechanoreceptors located in the whisker follicle. As with limb movements, whisker movements are reported by a set of receptors that is separated from those sensing touch [ 3].

Whisker afferents ascend via the thalamus in three parallel pathways: the lemniscal pathway ascends via the dorsomedial (dm) sector of the ventral posteromedial nucleus (VPM) (VPMdm), the paralemniscal ascends via a rostral sector of the posterior complex (POm), and a recently discovered pathway ascends via the ventrolateral sector of the VPM (VPMvl) [ 4]. We refer to the recently discovered pathway as “extralemniscal” to denote its path, which emerges from paralemniscal nuclei in the brainstem and ascends in parallel to the lemniscal and paralemniscal pathways [ 4] (we thank P.M. Knutsen for this suggestion). The paralemniscal, extralemniscal, and lemniscal pathways appear to be trigeminal analogs of the spinal spinothalamic, neospinothalamic, and dorsal column–lemniscal pathways [ 5], respectively. These pathways convey their information to different targets [ 4, 6, 7], which close the sensory-motor loop at different levels of brain hierarchy [ 8, 9], with the lemniscal involving the highest, the extralemniscal a lower, and the paralemniscal a still lower level. The lemniscal and paralemniscal pathways differ considerably in their responses to stimuli applied passively to stationary whiskers [ 10– 13]. However, information about the signals conveyed by these pathways, and by the extralemniscal one, during active touch is lacking [ 4].

We combined active whisking with controlled stimulus application and accurate localization of recording sites by employing artificial whisking in anesthetized rats [ 3, 14– 16]. With this method, whiskers are moved forward by their muscles, and thus whisker–object interaction mimics that which occurs naturally, i.e., forces are applied both to the whisker's follicle and to the whisker's shaft. In contrast, when stimulating passive whiskers, i.e., when the object moves an otherwise stationary whisker, forces are applied only to the whisker's shaft. Using this artificial active whisking method, we previously identified three types of active-touch signals (whisking, touch, and combined whisking–touch) sent by trigeminal ganglion (TG) neurons to the brain [ 3]. Here, we used the same method to examine conveyance of active-touch signals via the thalamus.

## Results

### Anatomical Borders

**Figure 1:**
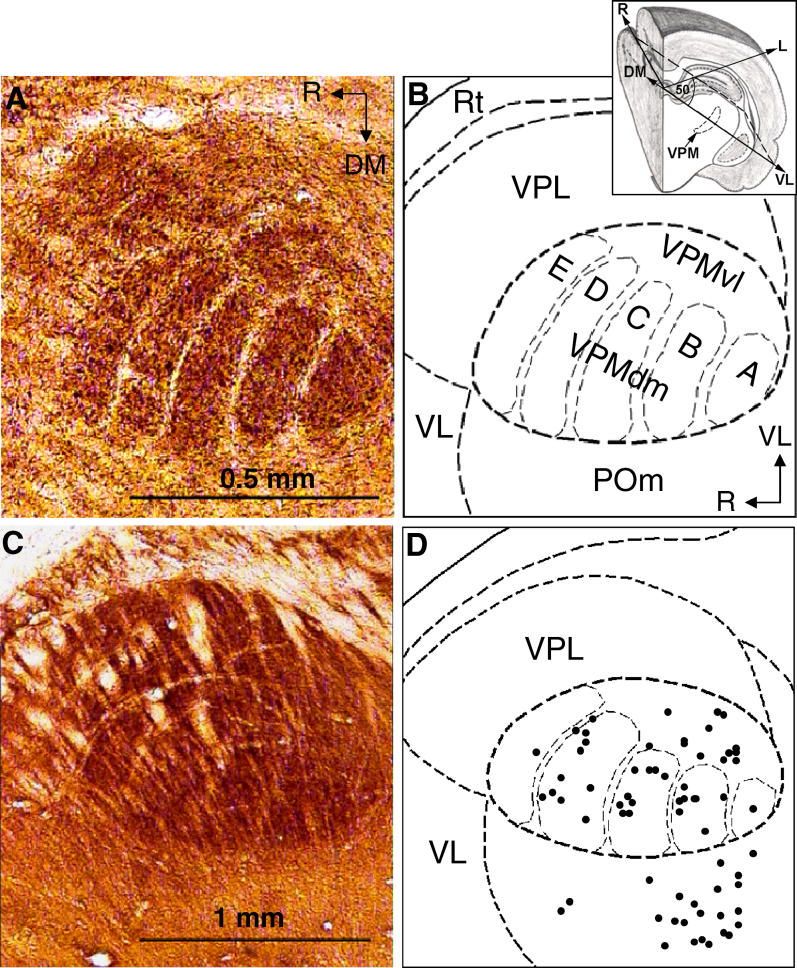
Thalamic Nuclei and Recording Sites (A and C) Oblique sections, dorsomedial to ventrolateral at 50° clockwise to the horizontal plane when the right hemisphere was viewed rostrally (Inset in [B]), through the thalamus of a young (postnatal day 7) (A) and an adult (340 g) (C) rat stained for CO. Depths from bregma were 2.9 mm for the dorsomedial and 3.6 mm for the ventrolateral end of the section shown in (A) and 4.4 mm and 5.85 mm, respectively, for the section shown in (C). Scale bars indicate 0.5 mm (A) and 1.0 mm (C). Arrows: R, rostral; DM, dorsomedial. (B and D) Borders between thalamic nuclei shown in (A) and (C), respectively, determined according to Paxinos and Watson's Atlas [ 46], using horizontal planes between 5.10 to 6.60 mm from bregma. Black dots in (D) indicate the projection of all recording sites ( *n* = 67) on the oblique plane. A–E barrelloids corresponding to whisker rows A–E, respectively. Arrows: L, lateral; VL, ventrolateral. Rt, reticular nucleus; VL, ventrolateral nucleus; VPL, ventroposterolateral nucleus.

In thalamic slices, the border between VPM and POm is distinct in all planes of sectioning, due to a high contrast in several anatomical markers, including cytochrome oxidase (CO) [ 17]. However, the border between VPMdm and VPMvl is not distinct with standard section planes, i.e., coronal, sagittal, and horizontal [ 4, 18]. Thus, we explored non-standard section planes in both young (in which thalamic borders are in general more clear) [ 19] and adult rats. We found that a distinct border between VPMdm and VPMvl is visible in an oblique plane, from dorsomedial to ventrolateral, at 50° to the horizontal plane [ 19]. In this plane, the VPMdm–VPMvl border was salient in young rats ( Figure 1A and 1B), and still visible in adult rats ( Figure 1C and 1D). We used the anatomical scheme of Figure 1D as a canonical scheme for our thalamic recordings. Each recording site was determined by an electrolytic lesion and mapped onto the canonical scheme ( Figure 1D, black dots). The method we used for coordinate transformation is described in Figure S1.

### Specificity of Thalamic Responses

We examined the specificity of neuronal responses to active movement and touch in the three parallel trigeminal pathways by recording from 67 individual neurons located in their corresponding thalamic stations in urethane-anesthetized rats ( Figure 1D): POm ( *n* = 24), VPMvl ( *n* = 13), and VPMdm ( *n* = 30). The facial nerve was stimulated at 83 Hz for 100 ms to induce protraction (forward movement of all whiskers), and then left unstimulated for 100 ms to allow passive retraction. Repetitive whisking movements were thus induced at 5 Hz, which is within the natural whisking rate, in trains of 2 s and intertrain intervals of 3 s. In the movement path of the principal whisker of each recorded neuron, a pole of 2-mm diameter was presented vertically during touch blocks (consisting of 12–24 trains each), at 70%–90% of the whisker's length. No object was presented in free-air blocks.

**Figure 2:**
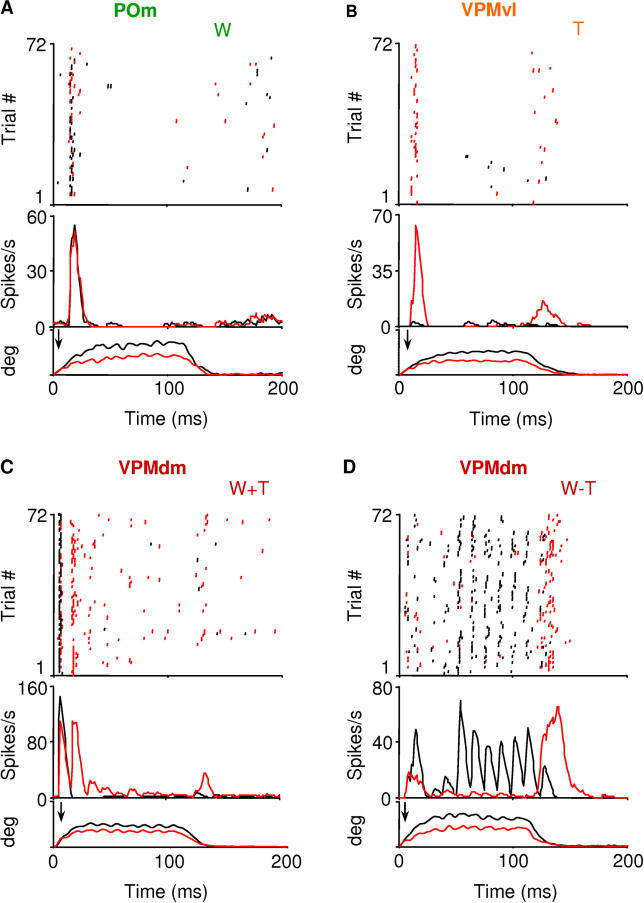
Responses of Individual Neurons during Steady State in the POm, VPMvl, and VPMdm Examples of responses of individual neurons during steady state are shown for the POm (A), VPMvl (B), and VPMdm (C) and (D) to whisking in air (black) and whisking against an object (red), relative to the time of protraction onset (t = 0). For each cell, the top graph depicts a raster display of single spikes accumulated from three randomly selected cycles from cycles 5–10. Middle graphs depict the PSTH computed for the entire steady-state period (cycles 5–10). Bottom graphs depict the horizontal angle of the principal whisker during a single cycle; ordinate denotes whisker angle, full scale = 50°, and traces were low-pass filtered at 160 Hz. Contact times, during touch trials, are indicated by arrows.

With moving whiskers, object localization would be ambiguous unless the brain contained an independent signal that described whisker motion. A pure movement (whisking) signal (W) would report whisking only, i.e., would report whisker movement in a consistent manner regardless of touch events during the movement. A pure touch signal (T) would report touch only. We found that a clear dissociation between W and T signals occurs in the thalamus, in which W and T signals are conveyed mainly by POm and VPMvl neurons, respectively. Most POm neurons responded to whisking movements independently of whether the whiskers contacted an object or not. For example, a neuron recorded from POm responded similarly ( Figure 2A, two top graphs) even though the whisker trajectory during touch (red) deviated upon contact from that in free air (black) ( Figure 2A, bottom graph). In contrast, most VPMvl neurons responded only when contacting an external object (see Figure 2B). VPMdm neurons exhibited combined whisking and touch signals, whose interactions were either additive (W + T) or subtractive (W − T), i.e., in which touch either added or subtracted spikes from the whisking response (see Figure 2C and 2D). In both free-air and touch conditions, tonic responses often contained a strong 83-Hz component (see rhythmic responses in Figure 2C and 2D), locked to the 83-Hz movement ripple (see whisker angle trajectories in Figure 2), similar to tonic responses of TG neurons [ 3]. Tonic responses were observed only in VPMdm, except one case in VPMvl (see response durations in Table S1). A period with reduced response between the phasic and tonic components, such as that exhibited in Figure 2D during whisking in air, was observed in seven VPMdm neurons (two W − T and five W + T) during either whisking in air or against an object.

**Figure 3:**
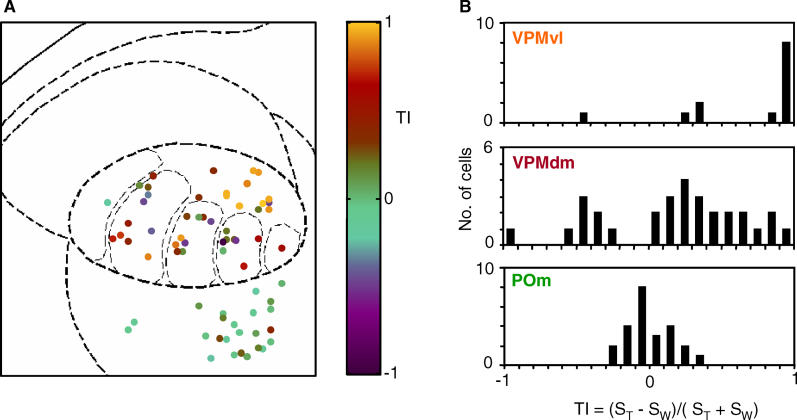
Distributions of Normalized Touch Responses in the Thalamus (A) TI value of each of the recorded neurons is indicated by a color code on its relative location in the canonical thalamic map defined in Figure 1D. (B) Distribution of TI in VPMdm ( *n* = 30), VPMvl ( *n* = 13), and POm ( *n* = 24).

Whisking and touch signals were quantified by measuring the response (spike count) of cells to whisking in air (S _W_) and to whisking against an object (S _T_) during the first 100 ms of each whisking cycle. These first 100 ms of each cycle contained only spikes generated during protraction, which comprised most of the spikes generated by our thalamic neurons in both whisking and touch conditions (see response durations in Table S1). Thalamic responses in all three nuclei exhibited a dynamic phase, lasting three to four whisking cycles, during which the response changed from cycle to cycle, followed by a steady-state phase during which the response remained stable (see Materials and Methods). The stable steady-state response, averaged over the last six cycles of each whisking train, was used to classify the type of thalamic response encountered. The touch component of the response was estimated as S _T_ − S _W_, i.e., the response during protraction in touch cycles minus the response during protraction in free air. Normalized touch responses (touch index [TI] = [S _T_ − S _W_]/[S _T_ + S _W_]) would be 0 for a cell conveying a pure whisking signal (i.e. response to whisking is the same with and without touch), 1 for a cell conveying a pure touch signal, and −1 for a cell whose whisking response is completely inhibited by touch. We classified cells as W if their S _T_ and S _W_ responses did not differ significantly and their |TI| < 0.2, T if their TI > 0.8, and WT otherwise (see Figure S2 for statistical justification of these thresholds). Distribution of the TIs of individual thalamic neurons across the thalamus ( Figure 3A) revealed a clear anatomical dissociation of W, T, and WT signals (see Table S1 for details): W signals (TI ˜ 0) are conveyed mostly (94%; 17/18 W cells) via the POm, T signals mostly (75%; 9/12 T cells) via VPMvl, and WT signals (W + T and W − T) mostly (70%; 26/37 WT cells) via VPMdm. Consistently, the distribution of the TIs in each of the thalamic nuclei ( Figure 3B) shows that POm neurons cluster around TI approximately 0, most VPMvl neurons cluster around TI approximately 1, and VPMdm neurons distribute bimodally, with most of the neurons exhibiting 0 < TI < 1 and fewer neurons exhibiting −1 < TI < 0.

### Comparison of Latency and Duration of Responses

**Figure 4:**
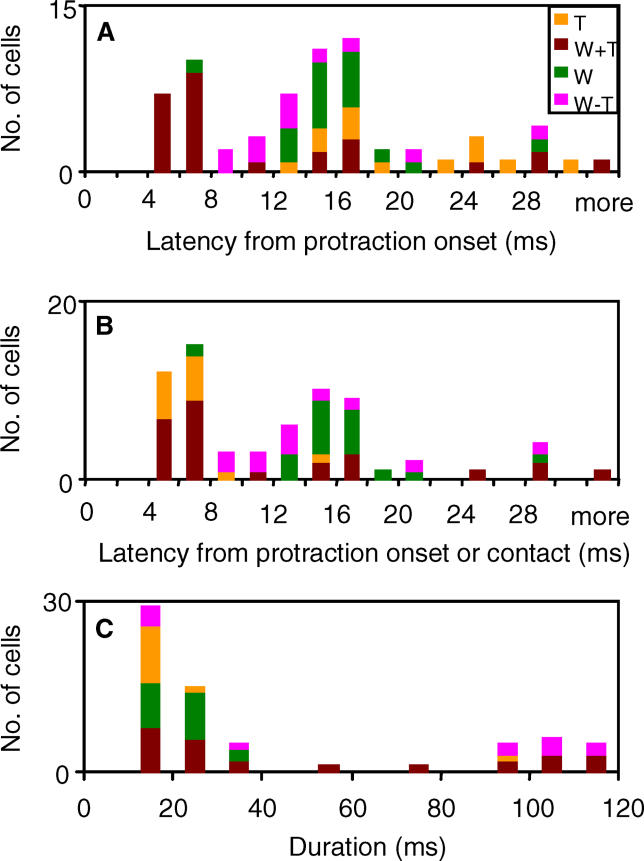
Distributions of Thalamic Latencies and Durations According to Response Type (A) Latencies from protraction onset to half-peak response of all thalamic neurons during touch trials. (B) Latencies from relevant stimulus (contact time for Touch neurons and protraction onset for the rest) to half-peak response of all thalamic neurons during touch trials. (C) Response durations of individual cells during touch trials, measured from the PSTH, as the period during protraction in which the response was above 0.1 of its maximum.

During touch cycles, latencies (from whisking onset to half-peak response) of W + T neurons (median = 6.7 ms) were significantly shorter than those of T (median = 17.6 ms) and W (median = 15.6 ms) neurons ( *p* < 0.006, non-parametric Mann-Whitney test) ( Figure 4A). Thus, W + T responses could not result from an integration of thalamic W and T responses. Latencies of T neurons from the time of contact were short (median = 6.7 ms), and comparable to the latencies of W + T neurons from whisking onset ( Figure 4B) ( *p* = 0.27, Mann-Whitney test). Interestingly, latencies of W − T cells (median = 12.4 ms) were longer than those of W + T neurons ( *p* = 0.05, Mann-Whitney test). Latencies also differed significantly across nuclei; POm neurons responded with longer latencies than VPMdm and VPMvl (from contact) ( *p* < 0.001, Mann-Whitney test), and VPMvl responded later than VPMdm neurons relative to whisking onset ( *p* = 0.04, Mann-Whitney test). The same relationships were observed when latencies were computed as delays from stimulus onset to the first spike in a cycle (see Table S1).

In these experiments, W and T responses were significantly shorter ( *p* < 0.001, paired *t* test) than the duration of protraction, lasting < 40 and 25 ms, respectively (W, 22 ± 7 ms; T, 16 ± 4 ms [excluding one outlier in VPMdm, whose duration was 92 ms]; their durations at half-peak were 13 ± 6 and 7 ± 1 ms, respectively) ( Figure 4C). Thus, W (POm) neurons mainly responded to the initial phase of protraction, when whisker velocity was highest, similar to most Whisking neurons of the TG [ 3]. T (VPMvl) neurons mainly reported contact onset, similar to TG Contact neurons [ 3]. In contrast, response durations of WT (W + T and W − T) neurons spanned two modes, one brief (< 40 ms; 21 ± 8 ms, 20/37 neurons) and one long (55–118 ms; 102 ± 16 ms, 17/37 neurons), which together covered the entire protraction phase ( Figure 4C). This bimodal distribution of WT response durations resembles that of TG Whisking/Touch neurons [ 3]. The distribution of response durations differed significantly between the three response types (W, T, WT; *p* < 0.02, Mann-Whitney test) and between the three nuclei ( *p* < 0.002, Mann-Whitney test).

## Discussion

**Figure 5:**
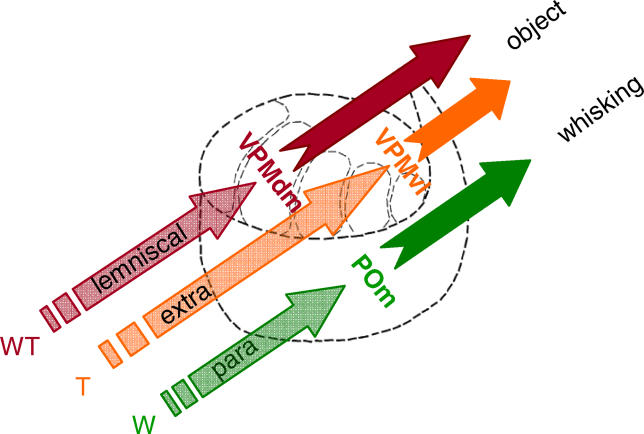
Proposed Scheme of Afferent Conduction of Active-Touch Signals Whisking signals (W) are conveyed by the paralemniscal pathway (para) via the POm and are proposed to involve whisking control. Touch signals (T) are conveyed by the extralemniscal pathway (extra) via VPMvl, and are proposed to involve processing of object location (“where”). Combined whisking–touch signals (WT) are conveyed by the lemniscal pathway via VPMdm, and are proposed to involve processing of object identity (“what”).

We showed here that the major active-touch signal conveyed in each of the three afferent pathways of the whisker system is different: whisking in the paralemniscal (via POm), contact in the extralemniscal (via VPMvl), and combined whisking–touch in the lemniscal (via VPMdm) pathway. The three afferent pathways did not respond synchronously. In each whisking cycle, VPMdm neurons, conveying the combined signal, fired first whereas POm and VPMvl neurons, conveying isolated whisking and touch signals, fired later. VPMdm also contained tonic responses that were absent in the other nuclei. All these observations, together with the known anatomy and physiology of the system, suggest that VPMdm responses did not result from a combination of signals transmitted by the POm and VPMvl. Moreover, the orthogonal response types of POm (W) and VPMvl (T) indicate that each of the three thalamic nuclei conveys a signal that could not result from a combination of signals transmitted by the other two nuclei. This, and the fact that similar response types (W, T, and WT) are exhibited by different classes of TG neurons, strongly suggest that the three thalamic nuclei are driven primarily by their afferent pathways. (W − T signals, whose latencies do allow intra-thalamic inhibition and/or cortical feedback [ 20] as primary drivers, might be an exception). Thus, our results suggest parallel afferent processing via the thalamus ( Figure 5).

Previously, we showed that POm neurons represent the temporal frequency of passive whisker movements by latency and spike count [ 12], and suggested that the POm is involved in temporal decoding of signals that encode whisker movement and of signals that encode the horizontal coordinate of object location [ 21, 22]. Our current data show that the POm does not convey contact information, and thus cannot resolve object location by its own. Taken together, our data now suggest that the POm is involved in temporal processing related to sensory-motor control of whisker movement, and that the POm and VPMvl together are involved in temporal processing of object location. In such processing, the POm would convey the reference signal and VPMvl the contact signal [ 8].

Why would sensory information flow in parallel pathways in this, or in other systems [ 10, 12, 23– 29]? Based on the anatomical and physiological data available, Bishop [ 5] suggested that parallel sensory pathways evolved in successive steps, each adding a larger fiber pathway, and incorporating successively higher brain areas to implement a novel function. Thus, Bishop suggested that the first spinal somatosensory pathway to evolve was the spinothalamic, followed by the neospinothalamic, and then the dorsal column–lemniscal. An order that is analogous to the paralemniscal, extralemniscal, and then lemniscal in the trigeminal system. The functional segregation reported here between these pathways, and the evidence indicating that these three pathways close the sensory-motor loop at different levels of brain hierarchy, raise the following sensory-motor hypothesis: The paralemniscal system is involved in a low-order motor-sensory-motor loop that controls whisking velocity and frequency in a servo-like manner [ 30], the extralemniscal system adds a higher level of control based on contact information and object location, and the lemniscal system adds the highest level of control so far, which is based on information related to object identity. This proposed functional segregation ( Figure 5) does not imply functional isolation; these parallel loops are expected to interact such that a higher loop uses, and builds upon, the processing performed by a lower loop. For example, the paralemniscal loop might interact with the brainstem loop [ 31] to optimize whisking control. Another example is object localization, in which contact timing (extralemniscal) must interact with whisking information (paralemniscal) to extract object location. Analysis of object identity requires interaction of detailed spatial information with information about whisker movement and contact [ 16, 32]. The high-resolution directional-selective spatial information [ 33, 34] together with whisking information (WT signals) conveyed by the VPMdm meet this requirement. Object-identity analysis also involves comparisons with memorized patterns; hence, it requires significant cortical involvement [ 35, 36], such as that exhibited by the lemniscal system. Thus, the paralemniscal, extralemniscal, and lemniscal parallel loops may have evolved sequentially, as suggested for parallel sensory pathways [ 5], by adding contact detection to movement control, and identity analysis to contact detection.

Our experimental paradigm utilized rats under general anesthesia, which affects response amplitude, latency, duration, and adaptation in the thalamus and cortex [ 11, 37– 39]. However, these effects are quantitative in nature and are expected to be similar for all thalamic neurons, and thus cannot account for the prominent differences in response types we report here. The state of thalamic and cortical neurons during the steady-state response phase, the phase used herein for response classification in anesthetized rats, is considered to be analogous to the state of thalamic and cortical neurons during exploratory whisking in awake rats [ 39– 41]. Consistently, during the steady state, thalamic neurons are hypothesized to function in their gating, signal-processing mode [ 42]. Nevertheless, under anesthesia, the intensity and nature of top-down effects, such as those affecting the thalamus directly, or indirectly [ 43, 44], are probably different; the efferent signals that control whisking are lacking; and the sensory-motor loops that control active touch [ 8] are practically opened. Moreover, behaving rats continuously control their whisking according to context and in reaction to contacts. Thus, although the basic segregation of response types observed here in anesthetized rats is expected to occur in awake ones, the exact behavior of thalamic neurons during active touch should be further studied in awake behaving rats.

## Materials and Methods

### Surgical and recording procedures

Experiments were performed on 40 male Albino Wistar rats weighing 200–300 g, using experimental protocols as previously described [ 3]. Briefly, surgery was performed under general anesthesia (urethane; 1.5 g/kg, intraperitoneally), with supplemental doses of anesthetic (10%) being administered when required. Atropine methyl nitrate (0.3 mg/kg, intramuscularly) was administered to prevent respiratory complications. Anesthetized animals were secured in a stereotaxic device (SR-6; Narishige, Tokyo, Japan), and their body temperature maintained at 37 °C. An opening was made in the skull overlying the right thalamus, and tungsten microelectrodes (0.5–1 MΩ; Alpha Omega Engineering, Nazareth, Israel) were lowered according to known stereotaxic coordinates of POm and VPM until units drivable by whisker stimulations were encountered. Up to four electrodes, spaced 0.33 mm from each other, were lowered in parallel in each recording session. Standard methods for single-unit recordings were used [ 3]. Single units were sorted by spike templates. Units were considered single only if they had homogenous spike shapes that did not overlap with other units or noise and if they exhibited refractory periods of > 1 ms in their autocorrelation histograms. Artifacts produced by electrical stimulation were isolated by an online spike-sorter (MSD-3.21; Alpha-Omega Engineering) and removed from unit recordings. Experimental procedures were approved by the Institutional Animal Care and Use Committee of The Weizmann Institute of Science.

### Experimental paradigms

We induced trains (5 Hz, 50% duty cycle, 2 s) of artificial whisking followed by intertrain intervals of 3 s in blocks of 12, 18, or 24 trains (trials) each. Artificial whisking was induced as described in [ 3]. In brief, the facial nerve was cut and its distal end mounted on a pair of silver electrodes. Bipolar, rectangular electrical pulses (0.5–4.0 V, 40 μs duration) were applied through an isolated pulse stimulator (Model 2100; A-M systems, Sequim, Washington, United States) at 83 Hz, the lowest frequency that still produces continuous whisker movement. Whisker movements were recorded at 1,000 frames/sec with a fast digital video camera (MotionScope PCI 1000; Redlake, San Diego, California, United States). Video recordings were synchronized with neurophysiological data with 1 ms accuracy [ 3, 45]. Blocks of free-air artificial whisking were interleaved with blocks of artificial whisking against an object positioned in front of the principal whisker, i.e., the whisker that produced the maximal response in the recorded cell during manual passive stimulations. The object was a vertical pole (2-mm diameter), positioned at three different horizontal distances from the resting position of the whisker; the distance of the object from the skin was 70%–90% of the whisker's length. Horizontal distances of the object from the resting position of the whisker ranged from 1 to 9 mm (median = 3 mm). Each of the four whisking conditions (free-air and three object positions) was repeated in at least two blocks, interleaved in time. Results of touch trials were averaged over all three object positions. In order to mimic as close as possible natural conditions, all the whiskers of the mystacial pad were left intact throughout an experiment. Thus, whiskers other than the principal whisker also contacted the object during whisker movement. However, we verified that the principal whisker was always the first whisker to contact the object during protraction. In the cases ( *n* = 11) in which other whiskers were between the principal whisker and the object, the other whiskers were moved rostral to the object prior to each block of trials.

### Histology and anatomical analysis

Procedures were identical to those previously employed [ 19]. Briefly, at the end of each recording session, electrolytic lesions were induced by passing currents (10 μA, 2 × 4s, unipolar) through the tips of the recording electrodes. The brains were then removed, fixed, sliced coronally, and stained for CO. Lesions located in the thalamus could be clearly seen (e.g., Figure S1). The coordinates of lesions in each rat were normalized to the size of the VPM of that rat, and translated from the coronal plane to an oblique plane (from dorsomedial to ventrolateral, at 50° to the horizontal plane [ 19]) and placed on a canonical map of the thalamus on that plane ( Figure 1D) (see Figure S1 for a detailed description of the coordinate transformation process). Sessions in which recording sites could not be determined were excluded from analysis.

### Analysis of whisking and neuronal data

Of the 97 individual neurons recorded, the recording sites of 14 could not be accurately localized, five did not respond to our stimulation paradigm, three responded only during retraction, and eight exhibited nonstationary behavior during the recording session. These 30 neurons were excluded from analysis, leaving 67 neurons in the dataset to be analyzed. Trajectories of whisker movements were analyzed offline, using a semi-automatic image processing software [ 45]. Whisking onset time was determined from the video records as the time at which the whiskers started moving. Raster plots and peri-stimulus time histograms (PSTHs; 1-ms bins, smoothed by convolution with a triangle of area 1 and a base of 10 ms) were computed and examined for all trains of each cell. Average response latencies were computed from PSTHs as the delay from certain events (protraction onset or contact) to half-peak response. Responses were analyzed during steady-state periods. We selected cycles 5–10 as those cycles in which virtually all thalamic neurons exhibited stabilized responses. This selection was based on the following observations. The intertrial variability (variance/mean) of response spike counts stabilized, on average, on cycle 1 for POm neurons and cycle 5 for VPM neurons. The mean cycle-to-cycle difference of four response variables (spike count/cycle, PSTH amplitude, latency to half-peak, and delay to first spike) stabilized at 0 for all three nuclei prior to cycle 5, except for spike count/cycle in the VPMdm, which stabilized on cycle 6.

## Supporting Information

Figure S1 Transformation of Recording Coordinates from Coronal to Oblique Plane (A1–A3) Coronal sections through the thalamus containing lesions (arrows) in the VPMvl (A1), VPMdm (A2), and POm (A3). Slices were counted starting from the rostral end of the VPM. “Slice x/y” indicates that the center of the lesion was found in slice no. x, out of total y slices that spanned the rostrocaudal length of the VPM in that rat.
(B) Normalization of recording site in the coronal plane. Each lesion is characterized by a ratio of two distances measured along the 50° dorsomedial-to-ventrolateral slope; S _i_, the distance of the lesion from the border between the POm and VPM, and D _i_, the distance between the POm/VPM border to the VPM/VPL border.
(C) Re-mapping of recording sites on the oblique plane. The rostrocaudal coordinate is determined by the normalized sequential number of the coronal slice; the normal rostrocaudal length of the VPM is set to 20 coronal slices. Thus, for example, slice 12 out of 18 is transformed to 20 × 12/18 = 13.3. The dm-vl coordinate of a site i (i = 1,2,3) is si = d _i_ × S _i_/D _i_, where d _i_ is the POm/VPM to VPM/VPL distance in the oblique plane at the rostrocaudal coordinate of site i, and S _i_/D _i_ is the ratio obtained for that site in the coronal section (B). s _1–3_ and d _1–3_ correspond to the lesions depicted in A1–A3. Scale bars indicate 1 mm in all panels.
(2.2 MB DOC)

Figure S2 Classification of Response Types Based on Steady-State Responses: Statistical Significance and Criteria (A) For each cell, the probability (one-tailed *t* test, across all steady-state cycles) that it did not respond to whisking in air (S _W_ = 0) is depicted as a function of its TI (i.e., the normalized touch responses during protraction). Green indicates POm cells; brown, VPMdm cells; and orange, VPMvl cells.
(B) For each cell, the probability (two-tailed *t* test) that its responses to whisking in air (S _W_) and whisking against an object (S _T_) were identical is depicted, as a function of its TI.
(C) Distribution of TI in the trigeminal thalamus ( *n* = 67).
Based on these data, cells with TI > 0.8 were classified as T (Touch) cells (dotted box in [A]). Cells with |TI| < 0.2 and *p*(S _W_ = S _T_) > 0.05 were classified as W (Whisking) cells (dotted box in [B]).
(115 KB DOC)

Table S1 Response Types, Magnitudes, Latencies, and Durations in Each Thalamic Nucleus (57 KB DOC)

## Abbreviations

CO: cytochrome oxidase
POm: whisker-responsive rostral sector of the posterior complex of the thalamus
PSTH: peri-stimulus time histogram
S _T_: response (spike count/cycle) of cells to whisking against an object during protraction
S _W_: response (spike count/cycle) of cells to whisking in air during protraction
T: touch signal
TG: trigeminal ganglion
TI: touch index (normalized touch response)
VPM: ventroposteromedial nucleus of the thalamus
VPMdm: dorsomedial VPM
VPMvl: ventrolateral VPM
W: whisking signal
