# Supplementary material for: Parallel Thalamic Pathways for Whisking and Touch Signals in the Rat
Source: PLoS Biol. 2006 Apr 18;4(5):e124. doi: 10.1371/journal.pbio.0040124 (PMC1436027; doi:10.1371/journal.pbio.0040124)
Supplement: Supplementary file 1 [file pbio.0040124.sg001.doc]

**s_1_**

**s_2_**

**s_3_**

**d_3_**

**d_2_**

**d_1_**

**S_i_**

**D_i_**

**Figure S1.** Transformation of recording coordinates from coronal to oblique-horizontal plane. (A1-3) Coronal sections through the thalamus containing lesions (arrows) in the ventrolateral VPM (A1), dorsomedial VPM (A2) and POm (A3). Slices where counted starting from the rostral end of the VPM. "Slice x/y" indicates that the center of the lesion was found in slice no. x, out of total y slices that spanned the rostrocaudal length of the VPM in that rat. (B**)** Normalization of recording site in the coronal plane. Each lesion is characterized by a ratio of 2 distances measured along the 50˚ dorsomedial-to-ventrolateral slope; S_i_, the distance of the lesion from the border between the POm and VPM, and D_i_, the distance between the POm/VPM border to the VPM/VPL border. (C) Re-mapping of recording sites on the oblique-horizontal plane. The rostro-caudal coordinate is determined by the normalized sequential number of the coronal slice; the normal rostro-caudal length of the VPM is set to 20 coronal slices. Thus, for example, slice 12 out of 18 is transformed to 20*12/18 = 13.3. The dm-vl coordinate of a site i (i=1,2,3) is si = d_i_ * S_i_/D_i_, where d_i_ is the POm/VPM to VPM/VPL distance in the oblique-horizontal plane at the rostrocaudal coordinate of site i, and S_i_/D_i_ is the ratio obtained for that site in the coronal section (B). s_1-3_ and d_1-3_ correspond to the lesions depicted in A1-3. Scale bars are 1 mm in all panels.
