# Supplementary material for: Parallel Thalamic Pathways for Whisking and Touch Signals in the Rat
Source: PLoS Biol. 2006 Apr 18;4(5):e124. doi: 10.1371/journal.pbio.0040124 (PMC1436027; doi:10.1371/journal.pbio.0040124)
Supplement: Supplementary file 2 [file pbio.0040124.sg002.doc]

**Figure S2.** Classification of response types based on steady-state responses: statistical significance and criteria**.** (A) For each cell, the probability (one-tailed t-test, across all steady-state cycles) that it did not respond to whisking in air (S_W_=0) is depicted as a function of its TI [Touch Index, i.e., the normalized touch responses during protraction]. (B) For each cell, the probability (two-tailed t-test) that its responses to whisking in air (S_W_) and whisking against an object (S_T_) were identical is depicted, as a function of its TI. (C) Distribution of TI in the trigeminal thalamus (n=67). Green, POm cells; brown, VPMdm cells; orange, VPMvl cells.

Based on these data, cells with TI>0.8 were classified as T (Touch) cells (dotted box in A). Cells with |TI|<0.2 and p(S_W_=S_T_)> 0.05 were classified as W (Whisking) cells (dotted box in B).
