# Supplementary material for: Parallel Thalamic Pathways for Whisking and Touch Signals in the Rat
Source: PLoS Biol. 2006 Apr 18;4(5):e124. doi: 10.1371/journal.pbio.0040124 (PMC1436027; doi:10.1371/journal.pbio.0040124)
Supplement: Supplementary file 3 [file pbio.0040124.st001.doc]

SUPPORTING INFORMATION

Table S1. Response types, magnitudes, latencies and durations in each thalamic nucleus.

|  | | Cell type^a^ | | | |
| --- | --- | --- | --- | --- | --- |
| Thalamic region | | W-T | W | W+T | T |
|  | | Number of cells | | | |
| POm |  | 2 | 17 | 5 |  |
| VPMdm |  | 8 | 1 | 18 | 3 |
| VPMvl |  | 1 |  | 3 | 9 |
|  | |  | | | |
|  | | Responses *(spikes per cycle, mean ± SD)* | | | |
| POm | S_W_^b^ | 0.49 ±0.40 | 0.36 ±0.10 | 0.36 ±0.21 |  |
|  | S_T_ | 0.32 ±0.27 | 0.34 ±0.10 | 0.58 ±0.28 |  |
|  | p | < 0.026 | > 0.1 | < 0.029 |  |
| VPMdm | S_W_ | 1.76 ±1.15 | 0.28 | 0.44 ±0.46 | 0.02 ±0.00 |
|  | S_T_ | 0.63 ±0.74 | 0.29 | 0.83 ±0.56 | 0.29 ±0.04 |
|  | p | < 0.001 | 0.890 | < 0.022^c^ | < 0.001 |
| VPMvl | S_W_ | 3.10 |  | 0.21 ±0.04 | 0.01±0.01 |
|  | S_T_ | 1.13 |  | 0.40 ±0.13 | 0.36 ±0.20 |
|  | p | 0.000 |  | < 0.001 | < 0.001 |

|  | | Cell type | | | |
| --- | --- | --- | --- | --- | --- |
| Thalamic region | | W-T | W | W+T | T |
|  |  | Latencies to half-peak(ms)^d^ | | | |
| POm |  | 17.5-20.3 (18.9)^e^ | 12.8-28.9 (15.8) | 14.3-28.5 (17.7) |  |
| VPMdm |  | 10.0-29.4 (12.2) | 7.4 | 4.5-54.9 (6.6) | 5.9-7.3 (7.0) |
| VPMvl |  | 9.7 |  | 5.7-6.7 (6.5) | 4.1-14.7 (6.3) |
|  | |  | | | |
|  | | Delay to 1^st^ spikes(ms)^d^ | | | |
| POm |  | 22-27 (25)^e^ | 15-41 (22) | 18-42 (30) |  |
| VPMdm |  | 11-30 (15) | 8 | 5-55 (7) | 6-9 (8) |
| VPMvl |  | 10 |  | 6-7 (7) | 5-16 (7) |
|  | |  | | | |
|  | | Duration(ms)^f^ | | | |
| POm |  | 19-30 (25)^e^ | 12-38 (20) | 15-38 (27) |  |
| VPMdm |  | 13-115(103) | 27 | 15-118 (63) | 15-92 (20) |
| VPMvl |  | 82 |  | 12-14 (12) | 11-23 (16) |

^a^Cells were classified as T if their normalized touch responses (TI) during protraction > 0.8, W if their S_T_ and S_W_ responses did not differ significantly and |TI|<0.2 (see Figure S1), W-T if S_T_ < S_W_ and W+T if S_T_ > S_W_.

^b^S_W_, spikes per free-air whisk; S_T_, spikes per touch whisk; p, ranges of p-values, computed for every cell, which indicates the probability that for that cell single-whisk responses in free-air and touch whisks belong to the same distribution [two-tailed t-test, number of whisks for each cell was 144 (n=54 cells), 216 (n=13), or 288 (n=3)].

^c^One outlier had p = 0.136 (see Figure S1)

^d^Delays and latencies are from contact for T neurons and from protraction onset for all other neurons.

^e^Range (median)

^f^Durations were measured from the PSTHs, as the period in which the response was above 0.1 of its maximum during protraction.
